# Relapse in stage I(E) diffuse large B‐cell lymphoma

**DOI:** 10.1002/hon.2487

**Published:** 2017-10-30

**Authors:** Marcel Nijland, Karin Boslooper, Gustaaf van Imhoff, Robbie Kibbelaar, Peter Joosten, Huib Storm, Eric N. van Roon, Arjan Diepstra, Hanneke C. Kluin‐Nelemans, Mels Hoogendoorn

**Affiliations:** ^1^ Department of Hematology, University Medical Centre Groningen University of Groningen Groningen the Netherlands; ^2^ Department of Pathology Pathology Friesland Leeuwarden the Netherlands; ^3^ Department of Hematology Medical Centre Leeuwarden Leeuwarden the Netherlands; ^4^ Department of Clinical Chemistry Medical Centre Leeuwarden CERTE KCL Leeuwarden the Netherlands; ^5^ Department of Clinical Pharmacy and Pharmacology Medical Centre Leeuwarden Leeuwarden the Netherlands; ^6^ Department of Pathology and Medical Biology, University Medical Centre Groningen University of Groningen Groningen the Netherlands

**Keywords:** CNS, diffuse large B‐cell lymphoma, limited stage, *MYC*, relapse, survival

## Abstract

Despite a general favourable outcome in limited stage diffuse large B‐cell lymphoma (DLBCL), relapses occur in about 10 to 20% of patients. Prognostic models only partially identify patients at risk for relapse. Moreover, it is not known whether the outcome after such a relapse is similar to the outcome after relapse in advanced stages. From January 2004 through December 2012, all newly diagnosed patients with stage I(E) DLBCL were retrospectively analysed from 2 clinical databases to investigate the relapse pattern and outcome in relation to initial treatment and clinical characteristics. In 126 patients (median age 64 years), histologically confirmed stage I(E) DLBCL was diagnosed. With a median follow‐up of 53 months (range 5‐132 months), 1 progressive disease and 18 relapses occurred. The 5‐year time to tumour progression and disease‐specific survival were 85% (95% CI 79‐91%) and 92% (95% CI 87%‐97%), respectively. We observed no significant difference in relapse localization, time to tumour progression, and disease‐specific survival between patients treated with abbreviated R‐CHOP plus involved field radiotherapy or with 6 to 8 cycles of R‐CHOP. Analysis of relapses showed relapse >5 years after initial treatment (late relapse) in 5 of 19 patients (26%). Six of 19 patients (32%) had central nervous system relapse. Three of 11 relapsed cases available for analysis (28%) showed an MYC translocation, suggesting an overrepresentation in the relapse group. Outcome of patients with a relapse was poor with a median survival after relapse of 8 months. Only 1 patient (5%) underwent successful autologous stem cell transplantation. To improve outcome in these patients, early identification of new biological factors such as a MYC translocation or a high risk for CNS dissemination might be helpful. Moreover, treatment of any relapse after stage I disease should be taken seriously. Salvage treatment should be similar to relapses after advanced DLBCL.

## 1 INTRODUCTION

Diffuse large B‐cell lymphoma (DLBCL) accounts for 25 to 30% of adult non‐Hodgkin lymphomas.^1^ Twenty‐five to 40% of patients present with limited stage disease, defined as stages I and II according to the Ann Arbor classification.

Until the beginning of this century, optimal treatment for limited stage DLBCL used to consist of 3 cycles of cyclophosphamide, doxorubicin, vincristine, and prednisone (CHOP) chemotherapy plus involved field radiotherapy (IFRT). This combined modality approach resulted in a significantly better overall survival (OS) than treatment with 8 cycles of CHOP alone.^2^ The addition of rituximab to CHOP has increased OS with 10‐15% in both limited stage and advanced stage DLBCL.^3^, ^4^, ^5^ Apparently, this became for many haematologists a reason to refrain from consolidation IFRT for patients with stage I and II.^6^ Although randomized controlled trials are lacking, a very large registry study covering >59,000 patients strongly suggested that combined modality therapy was associated with better OS, even in the rituximab era.^6^

Despite the generally favourable outcome, relapses still occur in 10 to 20% of patients with limited stage DLBCL and 5‐year OS ranges between 75% and 94%, which suggests that salvage of relapses is frequently unsuccessful.^6^, ^7^, ^8^, ^9^, ^10^ Clinical prognostic models only partially identify patients at risk for relapse.^2^, ^11^ Biological tumour characteristics such as cell of origin and especially presence of *MYC* translocation have prognostic significance in DLBCL.^12^, ^13^

We decided to analyse in an observational cohort study the relapses of patients with stage I(E) DLBCL focusing on (1) initial therapy (only R‐CHOP vs. combined modality treatment), (2) clinical characteristics and risk profile of the patient, (3) patterns of relapse, (4) if available the presence of *MYC* breaks, and (5) the final outcome after treatment. To this end, we used 2 large databases in the northern part of the Netherlands, thereby avoiding trial‐based selection and better approaching real life observations.

## 2 MATERIAL AND METHODS

### 2.1 Study design and patient identification

Clinical data on all consecutive patients with histologically confirmed stage I(E) DLBCL diagnosed during an 8‐year period from January 1, 2004 through December 31, 2012 were retrieved from 2 clinical databases from 5 medical centres and 1 academic medical centre. The combined databases are representative of the incidence, characteristics, and treatments of patients in the northern part of the Netherlands. Patients should have received at least 1 cycle of R‐CHOP. Primary coetaneous, central nervous system (CNS) large B‐cell lymphoma, primary mediastinal B‐cell lymphoma, and immunodeficiency lymphomas were excluded. At diagnosis, patients were staged by fludeoxyglucose positron emission tomography (^18^FDG PET) and/or computed tomography (CT) scans. The stage‐adjusted IPI and CNS IPI were used to stratify patients.^2^, ^14^ Pathological review was performed by experienced haematopathologists (RK and AD). Approval for this observational study was obtained from the Medical Ethics Review Committee from Medical Centre Leeuwarden. Informed consent was waived in accordance with Dutch regulations.

### 2.2 Treatment and follow‐up

Patients were categorized into 2 treatment regimens: combined modality treatment consisting of abbreviated R‐CHOP (3‐4 cycles) plus IFRT or R‐CHOP only (6‐8 cycles). The number of R‐CHOP cycles administered was registered for all patients. Staging was performed by ^18^FDG PET/CT scan in 59% of patients (n = 74) and by CT scan in 41% (n = 52). End of treatment response was assessed by ^18^FDG PET/CT scan in 75% of patients (n = 91) and by CT scan in 25% (n = 31). Tumour responses were classified as complete remission, partial response, stable disease, or progressive disease (PD) according to the International Working Group.^15^ In all patients, relapse was confirmed with ^18^FDG PET scan, CT scan, and/or magnetic resonance imaging. Histological confirmation of relapse (or in case of CNS localization multicolor flow cytometry of spinal fluid) was available in 68% (n = 13) of patients. Reasons for not performing histological conformation were early relapse (n = 4) and CNS localization (n = 2). In case of PD or relapse, the subsequent salvage treatment and response was retrieved from the clinical records. Early treatment‐related mortality was restricted to death during or ≤3 months after treatment. Relapses after 5 years were designated as late relapses.^16^, ^17^ Follow‐up was completed until December 2015.

### 2.3 MYC fluorescence in situ hybridization analysis

For evaluation of a *MYC* translocation, formalin‐fixed paraffin‐embedded tissue blocks were collected of the relapsed DLBCL cases. Interphase fluorescence in situ hybridization (FISH) was performed on 3‐μm‐thick whole tissue sections of the primary tumour as previously described by using Vysis break apart probes (Abbot Technologies).^18^

### 2.4 Statistical analysis

Duration of follow‐up was calculated for all patients alive. The primary endpoints were OS, disease‐specific survival (DSS), time to tumour progression (TTP), and survival after relapse. Overall survival was defined as time from diagnosis until death (from any cause), DSS as the time from diagnosis until death as a consequence of DLBCL, TTP as the time from diagnosis until relapse or progression, and survival after relapse as the time from relapse until death (from any cause).^15^ Survival curves were estimated according to the Kaplan‐Meier method. Between‐group differences in DSS and TTP were evaluated by using the log‐rank test. All categorical variables were expressed as counts and percentages. Where applicable, differences between groups were evaluated by chi‐square for binary variables and independent *t* tests for continuous variables. Cox regression was used for univariate analysis. Given the low incidence of events no multivariate analysis was performed. A 2‐tailed *P* value of less than.05 indicated statistical significance. All analyses were performed by using IBM SPSS Statistics version 22.

## 3 RESULTS

### 3.1 Clinical characteristics

A total of 126 patients with a median age of 64 years were eligible for analysis. At presentation, 41% (n = 50) of patients had a nodal localization; extranodal sites consisted of gastrointestinal (20%, n = 25), bone (10%, n = 13), and nasopharyngeal localization (11%, n = 14). Other sites encompassed the remaining 18%, i.e., testis (n = 7), thyroid (n = 5), breast (n = 5), and salivary glands (n = 2) (Figure SS1). High stage‐adjusted IPI was observed in 19% (n = 23) of patients. Central nervous system‐IPI 0‐1 (low risk), 2 to 3 (intermediate risk), and >3 (high risk) were observed in 52% (n = 66), 47% (n = 59), and 1% (n = 1), respectively.

### 3.2 Treatment

**Figure 1:**
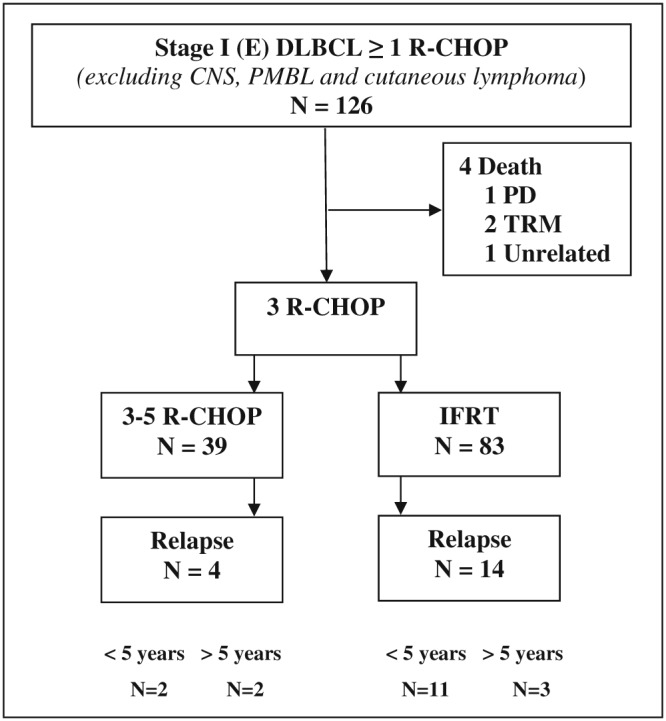
Schematic representation of the 126 patients with a stage I(E) diffuse large B‐cell lymphoma according to treatment regimen. One hundred twenty‐two patients completed therapy. Four patients died before completing 3 cycles of (R)CHOP. TRM, treatment‐related mortality; PD, progressive disease

Of the 126 patients 97% (n = 122) completed, at least 3 cycles of R‐CHOP were evaluable for comparison between treatment arms, e.g., combined modality (68%, n = 83) or R‐CHOP alone (32%, n = 39); see Figure 1 and Table 1. In patients receiving R‐CHOP, the number of cycles was reduced in 8% of patients (n = 3) because of previous tumour resection and treatment‐related toxicity. The cumulative dosage of IFRT was 30 to 40 Gray. All patients with testicular localization received CNS prophylaxis with intrathecal methotrexate. Patients with extranodal disease and those with elevated LDH more frequently received R‐CHOP only (*P* < .01 and *P* = .01, Table 1).

**Table 1:** Clinical characteristics of 122 patients with stage I(E) diffuse large B‐cell lymphoma who completed therapy according to treatment regimen

	Total (n = 122)	R‐CHOP (n = 39)	Abb R‐CHOP + IFRT (n = 83)	*P* value
Gender				.04
Male (%)	68 (56)	27 (69)	41 (49)	
Female (%)	54 (44)	12 (31)	42 (51)	
Median age (range)	64 (15‐87)	63 (15‐83)	66 (28‐87)	.43
Age < 60 (%)	48 (39)	16 (41)	32 (39)	.79
Age > 60 (%)	74 (61)	23 (59)	51 (61)	
Localization				<.01
Nodal (%)	50 (41)	8 (21)	42 (51)	
Extranodal (%)	72 (59)	31 (79)	41 (49)	
Performance				.37
WHO < 2 (%)	118 (97)	39 (100)	79 (95)	
WHO ≥ 2 (%)	4 (3)	0 (0)	4 (5)	
LDH				.01
Normal (%)	99 (81)	26 (67)	73 (88)	
Elevated (%)	23 (19)	13 (33)	10 (12)	
Stage‐adjusted IPI				.19
0‐1 (%)	99 (81)	29 (74)	70 (84)	
2‐3 (%)	23 (19)	10 (26)	13 (16)	

### 3.3 Clinical and biological characteristics of relapse

**Figure 2:**
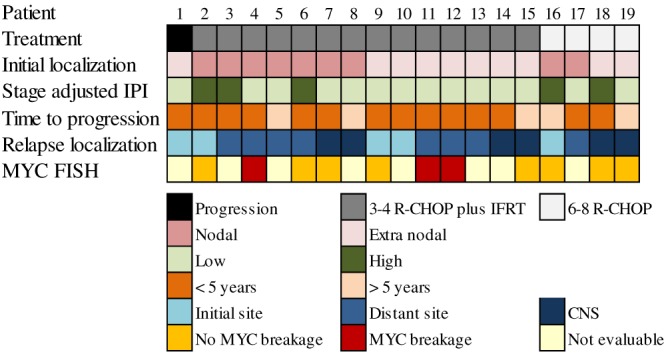
Schematic overview of clinical and biological characteristics of the 19 patients with a stage I(E) diffuse large B‐cell lymphoma who progressed during or relapsed after treatment. Patients are categorized according to the type of treatment received

One patient had PD during the first 3 cycles of R‐CHOP, and 18 patients experienced a relapse. Of these 18 patients, 28% of patients (n = 5) had a relapse more than 5 years after diagnosis: 3 of 14 patients treated with abbreviated R‐CHOP plus IFRT and 2 of 4 patients treated with R‐CHOP (p 0.52) (Figure 1). In 79% of cases (n = 15), the relapse/progression occurred at a site distant from the initial tumour localization. In 32% of cases (n = 6), this involved the CNS, with either meningeal and/or parenchymal localization. Two of the CNS relapses occurred in patients with a testicular lymphoma, despite CNS prophylaxis. In the remaining cases, the primary tumour had nodal (n = 2) and nasopharyngeal localizations (n = 2) (Figure 2). The initial calculated CNS‐IPI was low (n = 3) and intermediate (n = 3). In addition, 1 patient with initial nodal localization had a testicular relapse, another sanctuary site.

Of the 19 relapsed/progressive DLBCL, 58% of cases (n = 11) had tissue blocks with sufficient tumour material for *MYC*‐FISH analysis. In 28% of cases (n = 3), an *MYC* translocation could be demonstrated. All these *MYC* positive DLBCL were observed in patients with an early relapse. None of the evaluable CNS relapses had a *MYC* translocation (Figure 2).

### 3.4 Patient outcome

**Figure 3:**
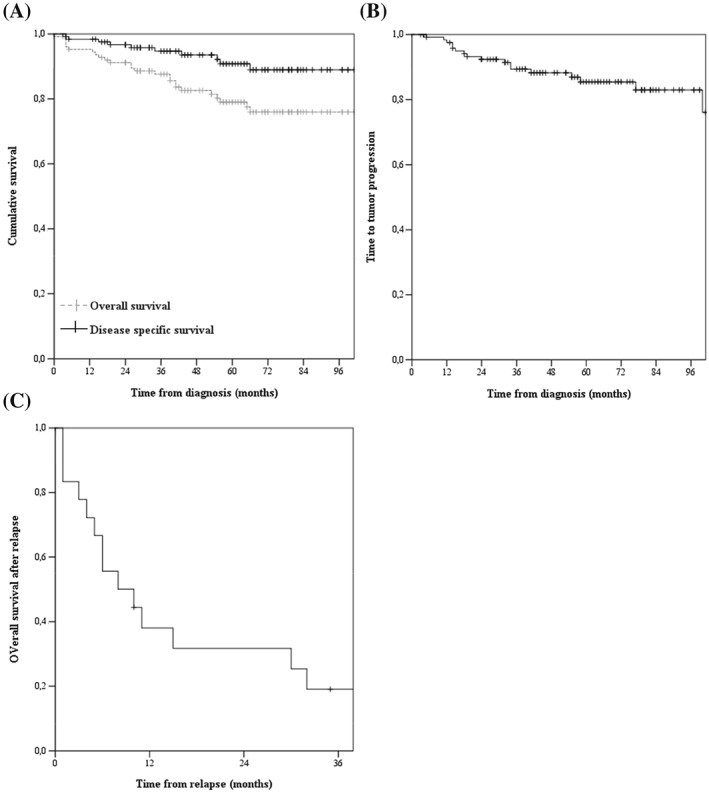
A, Kaplan Meier curves for overall survival (OS) and disease‐specific survival (DSS) of the 126 patients with a stage I(E) diffuse large B‐cell lymphoma (DLBCL). The 5‐year OS and DSS are 80 ± 6 and 92 ± 5%, respectively. B, Kaplan Meier curve for time to treatment failure (TTF) of the 126 patients with a stage I (E) DLBCL. At 5 years, the TTF was 85 ± 6%. C, Kaplan Meier curve for survival after relapse of 19 patients with a relapsed stage I(E) DLBCL. Median OS is 8.0 months

The median duration of follow‐up of the 126 patients was 53 months (range 5‐132). Twenty‐seven patients died (11 of relapse; 16 unrelated). The 5‐year TTP, DSS, and OS for the entire cohort were 85% (95% CI 79‐91%), 92% (95% CI 87%‐97%), and 80% (95% CI 73‐86%), respectively (Figure 3A and B). In univariate analysis of 122 patients completing therapy, age had the strongest association with shorter TTP (Table 2). Age > 60 years and elevated LDH, both composites of stage adjusted IPI, were associated with a shorter DSS (Table 2). Localization and treatment regimen were not found to be prognostic factors (Figure S2A). Univariate analysis showed that age > 60 years (HR 9.1, 95% CI 2.1‐38, *P* < .01), but not localization, LDH, or treatment regimen was associated with a shorter OS (Figure S2B).

**Table 2:** Univariate analysis of factors in time to tumour progression (TTP) and disease specific survival (DSS) of 122 stage I(E) diffuse large B‐cell lymphoma patients who completed therapy

	%	Hazard Ratio TTP	95% CI	*P* value	Hazard Ratio DSS	95% CI	*P* value
Gender							
Male	56	Reference			Reference		
Female	44	0.97	0.4‐2.5	0.94	0.94	0.3‐3.1	0.92
Age							
<60 years	39	Reference			Reference		
>60 years	61	4.1	1.2‐14	0.03	3.6	0.8‐17	0.06
Localization							
Nodal	41	Reference			Reference		
Extranodal	59	1.2	0.5‐3.0	0.72	1.2	0.3‐4.4	0.80
LDH							
Normal	81	Reference			Reference		
Elevated	19	2.0	0.7‐5.8	0.19	4.4	1.3‐15	0.02
Stage‐adjusted IPI							
0‐1	81	Reference			Reference		
2‐3	19	2.7	1.0‐7.5	0.05	4.5	1.3‐15	0.02
Treatment regimen							
Abb R‐CHOP + IFRT	68	Reference			Reference		
R‐CHOP	32	0.66	0.2‐2.0	0.47	2.0	0.6‐6.9	0.28

### 3.5 Treatment after relapse

The median age of relapsing patients was 77 years (range 41‐83). The median survival after relapse was 8 months (Figure 3C). Treatment of patients who relapsed consisted of rituximab, dexamethasone, cytarabine, and cisplatin salvage chemotherapy (16%, n = 3), retreatment with R‐CHOP (10%, n = 2), palliative chemotherapy (16%, n = 3), radiotherapy (32%, n = 6), and palliative care (26%, n = 5). Only 1 patient (5%) underwent high dose chemotherapy followed by autologous stem cell transplantation as part of the salvage regimen. A second remission was achieved in 3 of 5 (60%) patients receiving curative chemotherapy, in 1 patient (33%) treated with “intended” palliative chemotherapy, and in 2 patients (33%) treated with radiotherapy. One‐year survival after relapse for patients receiving salvage radiotherapy or chemotherapy was 53% (95% CI 27‐79%).

## 4 DISCUSSION

In this population‐based cohort study with a long median follow‐up, we followed all consecutive patients with newly diagnosed DLBCL stage I(E) during an 8‐year period. We observed no differences in relapse localization, TTP, and DSS between patients treated with abbreviated R‐CHOP plus IFRT and R‐CHOP only. Obviously, this was not a randomized comparison but a reflection of real‐life approaches. Patients with extranodal disease more frequently received R‐CHOP, reflecting physicians' choice to avoid radiotherapy‐induced toxicity. By looking into the characteristics of relapsed patients, we made several observations that offer a reason for the occurrence of relapses in these good risk patients.

Regardless of initial therapy, one‐third of relapses (26%) occurred more than 5 years after therapy. Although late relapses have been observed in the pre‐rituximab era,^17^ we and others observed these late relapses in patients with limited stage DLBCL treated with rituximab as well.^16^ Although clonal relationship in late relapses is established, the biology underlying the long interval remains unclear.^19^

Most relapses (73%, n = 15) arose at distant sites, indicative of good local tumour control with either abbreviated R‐CHOP plus radiotherapy or R‐CHOP. In nearly one‐third of relapses, there was CNS involvement. Because rituximab and CHOP have only limited activity in the CNS, it is unlikely that either of the treatment regimens will prevent the CNS relapses.^20^ Even when initial CNS prophylaxis with intrathecal MTX is provided, CNS relapses can occur as illustrated by 2 patients with lymphoma of the testes in our study.^21^ Recently, the CNS‐IPI as risk model for CNS relapse in patients with DLBCL was established to identify patients at highest risk for CNS relapse.^14^ In patients with a low‐risk CNS‐IPI, less than 1% showed a CNS relapse. However, as shown in our study, with stage I(E) DLBCL, 5% of 126 patients with initial low‐risk CNS‐IPI had a CNS relapse.

It was recently reported that in contrast to advanced stage DLBCL, the cell‐of‐origin is not prognostic in limited stage disease.^9^, ^13^ To impact prognosis of these good risk patients, a biomarker, such as *MYC* breaks, might be helpful.^12^ We found an *MYC* translocation in 3 of 11 evaluable relapsed cases. It is plausible that in the relapsed setting, 15 to 20% of DLBCL harbour an *MYC* translocation.^22^ Although the number of analysed patients is low, we found no *MYC* positive DLBCL in late relapses. Despite an increased risk of CNS dissemination in *MYC* positive DLBCL, no translocation was detected in the 2 evaluable CNS relapses.^23^

Combined analysis showed that two‐third of relapses could be assigned to either a late relapse, CNS relapse, or *MYC* positive DLBCL. In general, outcome of patients with relapsed or refractory DLBCL is very poor, with the exception of more favourable outcome in relapses more than 1 year after treatment.^24^ We observed a similar poor outcome in patients with relapsed stage I(E) DLBCL. This can partially be explained by the old age of the relapsed patients, limiting therapeutic options. Because the median age of patients with a DLBCL in the general population is 68 years, this is an observation in line with general practice. Furthermore, treatment options for relapsed DLBCL in the CNS are limited and patients with an *MYC* translocation tend to have a poor response to salvage chemotherapy.^25^, ^26^ Salvage therapy for a relapse after stage I(E) disease is not trivial and emphasizes the necessity to improve first line treatment in these patients as well.

## 5 CONCLUSIONS

Despite a general favourable outcome in stage I(E) DLBCL, 15% of patients relapsed despite previous R‐CHOP therapy and survival after relapse was short. Analysis of relapses showed that more than half of cases could be assigned to either a late relapse or CNS relapse. Interestingly, there is a suggestion that *MYC* positive DLBCL was overrepresented in the relapse group. Although numbers are small, our results emphasize the necessity to improve first line treatment in these patients as well. Any relapse after stage I disease should be taken seriously, and patients need similar intensive salvage therapy as after advance stage disease relapses.

## CONFLICT OF INTEREST

Not applicable.

## AVAILABILITY OF DATA AND MATERIALS

All relevant data and materials within this work are made available in this manuscript. Any additional information can be made freely available to any scientist on reasonable request.

## AUTHOR'S CONTRIBUTIONS

MN, KB, and MH contributed equally to designing research and writing the manuscript.

HK, ER, and MH established the databases. MN, GI, PJ, HS, HK, and MH contributed with clinical cases. RK and AD performed pathology review and MYC‐FISH analysis.

MN and KB analysed the results. GI, HK, and MH critically assessed and revised the manuscript. All authors read and approved the final manuscript.

## CONSENT FOR PUBLICATION

Not applicable.

## ETHICAL APPROVAL AND CONSENT TO PARTICIPATE

The study was conducted in accordance with the Declaration of Helsinki, International Conference on Harmonization guidelines, and relevant laws and regulations. Approval for this observational study was obtained from the Medical Ethics Review Committee from Medical Centre Leeuwarden. Informed consent was waived in accordance with Dutch regulations.

## Supporting information

Figure S1. Type of treatment of the 122 patients with a stage I(E) diffuse large B‐cell lymphoma (DLBCL) who completed therapy according to tumour localization. In nodal DLBCL, abbreviated R‐CHOP plus involved field radiotherapy is favoured over R‐CHOP. In extranodal DLBCL, nearly half of patients received R‐CHOP.
Figure S2. A. Overall survival (OS) of the 122 patients with a stage I(E) diffuse large B‐cell lymphoma (DLBCL) who completed therapy according to treatment regimen. The 5‐year OS of patients treated with abbreviated R‐CHOP plus involved field radiotherapy and R‐CHOP was 85% and 83%, respectively (p 0.47). B. Disease‐specific survival (DSS) for the 122 patients with a stage I(E) DLBCL who completed therapy according to treatment regimen. The 5‐year DSS of patients treated with abbreviated R‐CHOP plus involved field radiotherapy and R‐CHOP was 93% and 93%, respectively (p 0.32).

## References

[1] Stein H , Warnke RA , Chan WC et al. Diffuse large B‐cell lymphoma, NOS In Swerdlow SH , Camp E , Harris NL et al, editors. WHO Classification of Tumours of Haematopoeitic and Lymphoid Tissues. 4^th^ edition Lyon, France: International Agency for Resarch on Cancer (IARC); 2008:223‐237.

[2] Miller TP , Dahlberg S , Cassady JR , et al. Chemotherapy alone compared with chemotherapy plus radiotherapy for localized intermediate‐ and high‐grade non‐Hodgkin's lymphoma. NEJM. 1998;339:21‐26. 9647875 10.1056/NEJM199807023390104

[3] Pfreundschuh M , Kuhnt E , Trumper L , et al. CHOP‐like chemotherapy with or without rituximab in young patients with good‐prognosis diffuse large‐B‐cell lymphoma: 6‐year results of an open‐label randomised study of the MabThera international trial (MInT) group. Lancet Oncol. 2011;12:1013‐1022. 21940214 10.1016/S1470-2045(11)70235-2

[4] Coiffier B , Thieblemont C , Neste E , et al. Long‐term outcome of patients in the LNH‐98.5 trial, the first randomized study comparing rituximab‐CHOP to standard CHOP chemotherapy in DLBCL patients: a study by the groupe d'etudes des lymphomes de l'adulte. Blood. 2010;116:2040‐2045. 20548096 10.1182/blood-2010-03-276246 PMC2951853

[5] Sehn LH , Donaldson J , Chhanabhai M , et al. Introduction of combined CHOP plus rituximab therapy dramatically improved outcome of diffuse large B‐cell lymphoma in British Columbia. J Clin Oncol. 2005;23:5027‐5033. 15955905 10.1200/JCO.2005.09.137

[6] Vargo JA , Gill BS , Balasubramani GK , Beriwal S . Treatment selection and survival outcomes in early‐stage diffuse large B‐cell lymphoma: do we still need consolidative radiotherapy? J Clin Oncol. 2015;33:3710‐3717. 26261253 10.1200/JCO.2015.61.7654

[7] Persky DO , Unger JM , Spier CM , et al. Phase II study of rituximab plus three cycles of CHOP and involved‐field radiotherapy for patients with limited‐stage aggressive B‐cell lymphoma: southwest oncology group study 0014. J Clin Oncol. 2008;26:2258‐2263. 18413640 10.1200/JCO.2007.13.6929

[8] Tomita N , Takasaki H , Miyashita K , et al. R‐CHOP therapy alone in limited stage diffuse large B‐cell lymphoma. Br J Haematol. 2013;161:383‐388. 23432318 10.1111/bjh.12281

[9] Kumar A , Lunning MA , Zhang Z , Migliacci JC , Moskowitz CH , Zelenetz AD . Excellent outcomes and lack of prognostic impact of cell of origin for localized diffuse large B‐cell lymphoma in the rituximab era. Br J Haematol. 2015;171:776‐783. 26456939 10.1111/bjh.13766 PMC4715539

[10] Odejide OO , Cronin AM , Davidoff AJ , LaCasce AS , Abel GA . Limited stage diffuse large B‐cell lymphoma: comparative effectiveness of treatment strategies in a large cohort of elderly patients. Leuk Lymphoma. 2015;56:716‐724. 24913508 10.3109/10428194.2014.930853

[11] Norasetthada L , Nawarawong W , Bunworasate U , et al. Stage adjusted international prognostic index (st‐IPI) is a simple and better prognostic model in limited stage diffuse large B‐cell lymphoma (DLBCL): a nationwide multi‐institutional registry in Thailand. Blood. 2015;126(5030):

[12] Aukema SM , Siebert R , Schuuring E , et al. Double‐hit B‐cell lymphomas. Blood. 2011;117:2319‐2331. 21119107 10.1182/blood-2010-09-297879

[13] Scott DW , Mottok A , Ennishi D , et al. Prognostic significance of diffuse large B‐cell lymphoma cell of origin determined by digital gene expression in formalin‐fixed paraffin‐embedded tissue biopsies. J Clin Oncol. 2015;33:2848‐2856. 26240231 10.1200/JCO.2014.60.2383 PMC4554747

[14] Schmitz N , Zeynalova S , Nickelsen M , et al. CNS international prognostic index: a risk model for CNS relapse in patients with diffuse large B‐cell lymphoma treated with R‐CHOP. J Clin Oncol. 2016;34:3150‐3156. 27382100 10.1200/JCO.2015.65.6520

[15] Cheson BD , Pfistner B , Juweid ME , et al. International harmonization project on lymphoma. Revised response criteria for malignant lymphoma. J Clin Oncol. 2007;25:579‐586. 17242396 10.1200/JCO.2006.09.2403

[16] Stephens DM , Leblanc ML , Li H , et al. Continued risk of relapse independent of treatment modality in limited stage diffuse large B‐cell lymphoma: final and long‐term analysis of SWOG study S8736. J Clin Oncol. 2016;34:2997‐3005. 27382104 10.1200/JCO.2015.65.4582 PMC5012710

[17] Miller TP , LeBlanc M , Spier C . CHOP alone compared to CHOP plus radiotherapy for early stage aggressive non‐Hodgkin's lymphomas: update of the Southwest Oncology Group (SWOG) randomized trial. Blood. 2001;98:724a‐725a.

[18] Ventura RA , Martin‐Subero JI , Jones M , et al. FISH analysis for the detection of lymphoma‐associated chromosomal abnormalities in routine paraffin‐embedded tissue. J Mol Diagn. 2006;8:141‐151. 16645199 10.2353/jmoldx.2006.050083 PMC1867591

[19] de Jong D , Glas AM , Boerrigter L , et al. Very late relapse in diffuse large B‐cell lymphoma represents clonally related disease and is marked by germinal center cell features. Blood. 2003;102:324‐327. 12649152 10.1182/blood-2002-09-2822

[20] Zhang J , Chen B , Xu X . Impact of rituximab on incidence of and risk factors for central nervous system relapse in patients with diffuse large B‐cell lymphoma: a systematic review and meta‐analysis. Leuk Lymphoma. 2014;55:509‐514. 23741977 10.3109/10428194.2013.811239

[21] Vitolo U , Chiappella A , Ferreri AJ , et al. First‐line treatment for primary testicular diffuse large B‐cell lymphoma with rituximab‐CHOP, CNS prophylaxis, and contralateral testis irradiation: final results of an international phase II trial. J Clin Oncol. 2011;29:2766‐2772. 21646602 10.1200/JCO.2010.31.4187

[22] Thieblemont C , Briere J , Mounier N , et al. The germinal center/activated B‐cell subclassification has a prognostic impact for response to salvage therapy in relapsed/refractory diffuse large B‐cell lymphoma: a bio‐CORAL study. J Clin Oncol. 2011;29:4079‐4087. 21947824 10.1200/JCO.2011.35.4423

[23] Oki Y , Noorani M , Lin P , et al. Double hit lymphoma: the MD Anderson Cancer Center clinical experience. Br J Haematol. 2014;166:891‐901. 24943107 10.1111/bjh.12982

[24] Gisselbrecht C , Glass B , Mounier N , et al. Salvage regimens with autologous transplantation for relapsed large B‐cell lymphoma in the rituximab era. J Clin Oncol. 2010;28:4184‐4190. 20660832 10.1200/JCO.2010.28.1618 PMC3664033

[25] Ferreri AJ , Donadoni G , Cabras MG , et al. High doses of antimetabolites followed by high‐dose sequential chemoimmunotherapy and autologous stem‐cell transplantation in patients with systemic B‐cell lymphoma and secondary CNS involvement: final results of a multicenter phase II trial. J Clin Oncol. 2015;33:3903‐3910. 26282634 10.1200/JCO.2015.61.1236

[26] Herrera AF , Mei M , Low L , et al. Relapsed or refractory double‐expressor and double‐hit lymphomas have inferior progression‐free survival after autologous stem‐cell transplantation. J Clin Oncol. 2017;35:24‐31. 28034071 10.1200/JCO.2016.68.2740 PMC5455688

