# Supplementary figures and images for: Relapse in stage I(E) diffuse large B‐cell lymphoma
Source: Hematol Oncol. 2017 Oct 30;36(2):416–21. doi: 10.1002/hon.2487 (PMC5947732; doi:10.1002/hon.2487)

**Supplementary figure 1**


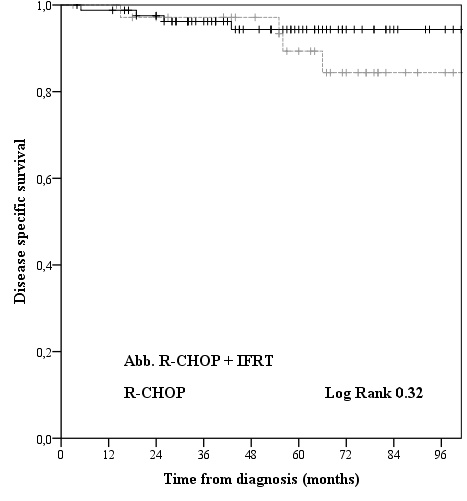

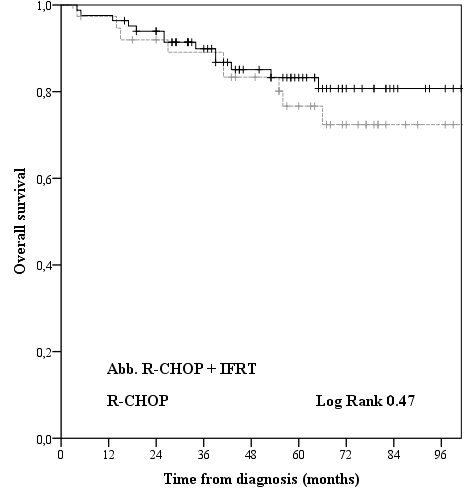
**Supplementary figure 2**

**A**

**B**

Supplement: Supplementary file 1 — Figure S1. Type of treatment of the 122 patients with a stage I(E) diffuse large B‐cell lymphoma (DLBCL) who completed therapy according to tumour localization. In nodal DLBCL, abbreviated R‐CHOP plus involved field radiotherapy is favoured over R‐CHOP. In extranodal DLBCL, nearly half of patients received R‐CHOP. Figure S2. A. Overall survival (OS) of the 122 patients with a stage I(E) diffuse large B‐cell lymphoma (DLBCL) who completed therapy according to treatment regimen. The 5‐year OS of patients treated with abbreviated R‐CHOP plus involved field radiotherapy and R‐CHOP was 85% and 83%, respectively (p 0.47). B. Disease‐specific survival (DSS) for the 122 patients with a stage I(E) DLBCL who completed therapy according to treatment regimen. The 5‐year DSS of patients treated with abbreviated R‐CHOP plus involved field radiotherapy and R‐CHOP was 93% and 93%, respectively (p 0.32). [file HON-36-416-s001.docx]
